# Evaluation of two-stage hepatectomy using portal vein embolization for colorectal liver metastasis: a retrospective nationwide cohort survey in Japan

**DOI:** 10.1097/JS9.0000000000001811

**Published:** 2024-06-13

**Authors:** Takayuki Shimizu, Taku Aoki, Mitsuru Ishizuka, Katsunori Sakamoto, Toru Beppu, Goro Honda, Kenjiro Kotake, Masakazu Yamamoto, Keiichi Takahashi, Itaru Endo, Kiyoshi Hasegawa, Michio Itabashi, Yojiro Hashiguchi, Yoshihito Kotera, Shin Kobayashi, Tatsuro Yamaguchi, Soichiro Natsume, Ken Tabuchi, Hirotoshi Kobayashi, Kensei Yamaguchi, Kimitaka Tani, Satoshi Morita, Masaru Miyazaki, Kenichi Sugihara, Yoichi Ajioka

**Affiliations:** a Joint Committee for National Survey on Colorectal Liver Metastasis, Tokyo; b Department of Surgery, Institute of Gastroenterology, Tokyo Women’s Medical, Tochigi; c Department of Surgery, Tokyo Metropolitan Health and Hospitals Corporation Ohkubo Hospital, Tokyo; d Department of Surgery, Hepato-Biliary-Pancreatic Surgery Division, Graduate School of Medicine, The University of Tokyo; e Department of Surgery, Teikyo University School of Medicine Tokyo; f Department of Surgery, Tokyo Metropolitan Cancer and Infectious Diseases Center Komagome Hospital, Tokyo; g Department of Pediatrics, Tokyo Metropolitan Cancer and Infectious Diseases Center Komagome Hospital, Tokyo; h Department of Gastrointestinal Chemotherapy, Cancer Institute Hospital of Japanese Foundation for Cancer Research, Tokyo; i International University of Health and Welfare, Narita Hospital, Tokyo; j Tokyo Medical and Dental University, Tokyo; k Department of Hepato-Biliary-Pancreatic Surgery, Dokkyo Medical University, Tochigi; l Department of Surgery, Sano City Hospital, Sano, Tochigi; m Department of Surgery, Utsunomiya Memorial Hospital, Utsunomiya, Tochigi; n Department of Hepato-Biliary-Pancreatic and Breast Surgery, Ehime University Graduate School of Medicine, Ehime; o Department of Surgery, Yamaga City Medical Center, Kumamoto; p Department of Gastroenterological Surgery, Yokohama City University Graduate School of Medicine, Kanagawa; q Department of Surgery, Teikyo University Hospital, Kanagawa; r Department of Hepatobiliary and Pancreatic Surgery, National Cancer Center Hospital East, Chiba; s Department of Biomedical Statistics and Bioinformatics, Graduate School of Medicine, Kyoto University, Kyoto; t Division of Molecular and Diagnostic Pathology, Graduate School of Medical and Dental Sciences, Niigata University, Niigata, Japan

**Keywords:** two-stage hepatectomy, colorectal liver metastasis, portal vein embolization

## Abstract

**Introduction:**

Two-stage hepatectomy (TSH) enables patients to undergo surgery for colorectal liver metastasis (CRLM), which one-stage hepatectomy cannot remove. Although the outcome of TSH has been reported, there is no original report from Japan. The aim of this retrospective study was to evaluate the outcome of TSH in Japanese patients with CRLM.

**Methods:**

The authors conducted a retrospective cohort study using the nationwide database that included clinical information of 12 519 patients treated with CRLM between 2005 and 2017 in Japan. The primary outcome measure was overall survival. The second outcome measure was progression-free survival. Fisher’s exact test, χ^2^ test and Mann–Whitney U test were conducted to examine an intergroup difference. Univariate and multivariate analyses were performed using Cox regression model. Survival analysis was performed by Kaplan–Meier method and log-rank test.

**Results:**

Of the database, 53 patients undergoing TSH using portal vein embolization (PVE) were identified and analyzed. Their morbidity and in-hospital mortality rates at the second hepatectomy were 26.4% and 0.0%. The mean observation period was 21.8 months. The estimated 1-, 3- and 5-year overall survival rate were 92.5%, 70.8% and 34.7%. Multivariate analyses showed that more than 10 liver nodules significantly increased the mortality risk by 4.2-fold (95% CI 1.224–14.99, *P*= 0.023). Survival analysis revealed that repeat hepatectomy for disease progression after TSH was superior to chemotherapy in overall survival (mean: 49.6 vs. 18.7, months, *P*= 0.004).

**Conclusion:**

In the Japanese cohort, TSH was confirmed to be a safety procedure with an acceptable survival outcome. More than 10 liver nodules may be a predictor for unfavorable outcomes of patients with CRLM undergoing TSH. Furthermore, repeat hepatectomy can be a salvage treatment for resectable intrahepatic recurrence after TSH.

## Introduction

Highlights

- Fifty-three patients with colorectal liver metastases who had undergone two-stage hepatectomy combined with portal vein embolization were identified from a nationwide database in Japan.
- Significant clinical factors associated with unfavorable survival outcomes were investigated.
- The morbidity and in-hospital mortality rate at the second hepatectomy were 26.4% and 0.0%, respectively. The estimated 1-, 3-, and 5-year overall survival rates were 92.5%, 70.8% and 34.7%, respectively.
- Multivariate analyses identified the presence of more than 10 liver nodules as being associated with unfavorable survival outcomes.

Colorectal cancer (CRC) is the third incidence and second most common cause of cancer mortality in the world^1^. Colorectal liver metastasis (CRLM) is the most common site of distant metastasis, and ~15–25% of patients with CRC have CRLM at the diagnosis of primary cancer^2^. Five-year survival rate of patients with metastatic CRC is less than 20%^3^. Because 5-year survival rate of hepatectomy for CRLM is 44.2–59.8%^4–8^, hepatectomy is recommended for resectable CRLM.

Two-stage hepatectomy (TSH) was developed as a safe procedure to address concerns about postoperative liver failure in cases of initially unresectable CRLM in 2000^9^. TSH involves two surgical processes: (1) The less advanced liver lobe is cleaned of its metastasis, and portal vein embolization (PVE) or ligation for the more advanced liver lobe is performed to grow remnant liver in the first hepatectomy, (2) Subsequently, liver lobectomy is conducted for the more advanced lobe in the second hepatectomy^10^. TSH provides an opportunity for patients with unresectable CRLM to undergo curative treatment.

TSH is considered as a safe procedure with 0.0–7.0% of mortality within 90 days^11–14^. Five-year survival rate after TSH was reported to be 23.0–44.2%^14–17^. However, 8.8–33.1% of patients didn’t complete TSH due to disease progression and insufficient growth of the remnant liver^11–14^. Total number of liver nodules, extrahepatic metastasis, major complications at the second surgery, repeat surgery for recurrence and RAS mutation were prognostic factors in patients with CRLM undergoing TSH^11,14,15,18^. While TSH for unresectable CRLM is increasing in Japan, original research of TSH in Japan is not yet established. This study aims to evaluate surgical outcomes for patients with CRLM undergoing TSH in Japan.

## Methods

### Ethics

This study was approved by the institutional review board. An opting-out method was used to obtain patient consent. This study was reported in line with the STROCSS guidelines^19^, Supplemental Digital Content 1, http://links.lww.com/JS9/C745. The study was performed in accordance with the Declaration of Helsinki.

### Patient selection

This retrospective cohort study was conducted by “The Joint Committee for Nationwide Survey on CRLM” in Japan. The Committee has created multiple databases of patients who were newly diagnosed with CRLM. One database (early years) contains the retrospective data on 3820 patients treated between 2005 and 2007, with follow-up until 2013. Other databases (late years) includes the prospective data on 3525 patients treated from 2013 to 2014 and 5174 patients treated from 2015 to 2017, with survival monitoring until 2019. This study focused on patients who underwent TSH for CRLM across these databases. We excluded patients who did not undergo TSH and patients who underwent hepatectomy twice for CRLM diagnosed at different times were excluded from the study to minimize the study bias in patient selection.

### Explanation of patient characteristics

Age, BMI and largest tumor diameter were at the diagnosis. A total number of liver nodules was finally confirmed post-TSH. Patient status, indocyanine green retention rate at 15 min (ICGR15), prothrombin time, total bilirubin, albumin, carbohydrate antigen (CA19-9) and carcinoembryonic antigen (CEA), JHPBS nomogram score were assessed before the first hepatectomy^20^. The CA19-9 and CEA were analyzed with the standard value 37.0 U/ml and 5.0 ng/ml, respectively. Based on tumor-node-metastasis (TNM) classification (8th edition) by union for International Cancer Control, T and N factor were determined. RECIST version 1.1 criteria were used for the effect of preoperative chemotherapy evaluation as follows: partial response [PR] and stable disease [SD]^21^. Residual tumor status was assessed after the second hepatectomy as follows: R0 was no residual tumor, R1 was a microscopic residual tumor and R2 was a macroscopic residual tumor.

### Outcome measure

The primary outcome measure was overall survival (OS). OS was measured from the date of the first hepatectomy to the date of death or last follow-up. The second outcome measure was progression-free survival (PFS). PFS was measured from the date of the first hepatectomy to the date of death or disease progression after TSH. Disease progression after TSH were defined as tumor recurrence, residual tumor progression or death.

### Statistical analysis

The values of Tables are parentheses and percentages or mean and standard deviation. Univariate and multivariate analyses using Cox proportional hazards model for OS after the first hepatectomy were performed with calculation of the hazard ratio (HR) and 95% CI. Variables with *P* value less than 0.05 in univariate analysis were incorporated into multivariate analysis. The Kaplan–Meier method was used to calculate the mean estimated survival period and estimated 5-year survival rate. A log-rank test was used to compare OS and PFS. Intergroup differences were analyzed using Fisher’s exact test, χ^2^ test or Mann–Whitney *U* test, as appropriate. The cut-off values of the continuous characteristics were determined using a receiver operating characteristics (ROC) curve analysis for OS. *P* values less than 0.05 were deemed statistically significant. All statistical analyses were performed using the SPSS software program (Version 28.0; IBM Co) and EZR version 1.61 (Jichi Medical University)^22^.

## Results

**Figure 1:**
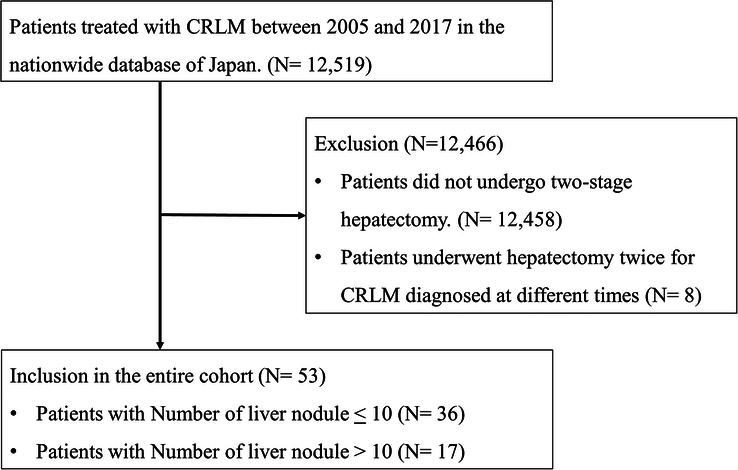
Selection of the study population. CRLM, colorectal liver metastasis.

Among 12 519 patients across the multiple databases, we excluded 12 458 who did not undergo TSH, and an additional 8 who underwent hepatectomy twice for CRLM diagnosed at different times were also excluded from this study (Fig. 1). Finally, 53 patients undergoing TSH were identified and reviewed. All patients were initially diagnosed with borderline resectable or unresectable CRLM. All patients completed TSH, underwent PVE at the first hepatectomy and liver lobectomy at the second hepatectomy. Additionally, 12 patients (22.6%) underwent colorectal surgery and their first hepatectomy simultaneously, while the other 41 patients (77.4%) underwent colorectal surgery before their first hepatectomy. The first hepatectomy was performed laparoscopically in 5 patients (9.4%), and 2 patients (3,7%) underwent the laparoscopic second hepatectomy. Supplementary Table 1, Supplemental Digital Content 2, http://links.lww.com/JS9/C746 exhibited underlying diseases of patients, indicating that 29 had no systemic disease, 23 had a mild systemic disease, 1 had a severe systemic disease and none were diagnosed with viral hepatitis.

Table 1 summarized the clinical characteristics in patients undergoing TSH. Of the entire patients, 5 underwent TSH in early years, and 48 underwent TSH in the late years. Forty-two patients received preoperative chemotherapy (79.2%). Of them, 29 patients received first-line regimen (54.7%) and 13 received more than second-line regimen (24.5%). First-line regimen of preoperative chemotherapy was FOLFOX-based therapy in 33 patients, 6 patients of CapeOX based therapy in 6, other regimen in one and unknown regimen in 2. Molecular-target therapy combined with FOLFOX or CapeOX was preoperatively demonstrated in 37 patients. Regarding the effect of preoperative chemotherapy, 23 patients had PR (43.4%), 15 had SD (28.3%) and 4 had unavailable data.

**Table 1:** Clinical characteristics of patients undergoing two-stage hepatectomy using portal vein embolization for colorectal liver metastasis.

Characteristics	Entire cohort (*n*=53)	No. nodules≤ 10 (*n*=36)	No. nodules> 10 (*n*=17)	*P*
Age (years), mean (s.d)^a^	60 (9)	66 (6)	55 (9)	0.015
BMI (kg/m^2^), mean (s.d)^a^ ^b^	21.3 (2.9)	21.9 (2.7)	22.3 (2.5)	0.475
Sex ratio (M:F)	27:26	20:16	7:10	
Timing of liver metastasis, *n* (%)				
Metachronous	1 (1.9)	0	1 (6.2)	0.142
Synchronous	52 (98.1)	36 (100.0)	15 (93.8)	
No. liver nodules, mean (s.d)^a^	11 (10)	6 (2)	23 (12)	<0.001
Largest tumor diameter (cm), mean (s.d)^a^	5.2 (3.8)	4.8 (3.8)	4.7 (4.2)	0.529
Primary tumor site, *n* (%)				
Colon	31 (58.5)	20 (55.6)	11 (64.7)	0.918
Rectum	13 (24.5)	9 (25.0)	4 (23.5)	
Unknown	9 (17.0)	7 (19.4)	2 (11.8)	
Primary tumor differentiation, *n* (%)				
Well	11 (20.8)	9 (25.0)	2 (11.8)	0.692
Moderate	29 (54.7)	20 (55.6)	9 (52.9)	
Poor or others	6 (11.3)	4 (12.2)	2 (11.8)	
Unknown	7 (13.2)	3 (8.3)	4 (23.5)	
T factor of primary tumor, *n* (%)				
T2	2 (3.8)	1 (2.5)	1 (5.9)	0.207
T3	34 (64.2)	23 (57.5)	11 (64.7)	
T4	12 (22.6)	11 (27.5)	1 (5.9)	
Not available	5 (9.4)	5 (12.5)	4 (23.5)	
Lymph node metastasis of primary tumor, *n* (%)				
N0	18 (34.0)	13 (36.1)	5 (29.4)	0.886
N1	20 (37.7)	13 (36.1)	7 (41.2)	
N2	15 (28.3)	10 (27.8)	5 (29.4)	
Lymphatic invasion of primary tumor, *n* (%)				
ly0	24 (45.3)	16 (44.4)	8 (47.0)	0.164
ly1	10 (18.9)	8 (22.2)	2 (11.8)	
ly2	14 (26.4)	7 (19.4)	7 (41.2)	
ly3	5 (9.4)	5 (13.9)	0 (0.0)	
Venous invasion of primary tumor, *n* (%)				
v0	14 (26.4)	8 (22.2)	6 (35.4)	0.451
v1	18 (34.0)	14 (38.9)	4 (23.5)	
v2	15 (28.3)	11 (30.6)	4 (23.5)	
v3	6 (11.3)	3 (8.3)	3 (17.6)	
KRAS mutation of primary tumor, *n* (%)				
Yes	12 (22.6)	8 (22.2)	4 (23.5)	0.493
No	22 (41.5)	12 (33.4)	10 (58.8)	
Unknown	19 (35.8)	16 (44.4)	3 (17.6)	
ICGR15 (%), mean (s.d)^a^ ^b^	12 (10)	11 (6)	15 (17)	0.599
Prothrombin time (%), mean (s.d)^a^ ^b^	95 (15)	99 (18)	87 (8)	0.339
Total bilirubin (mg/dl), mean (s.d)^a^ ^b^	0.6 (0.3)	0.7 (0.3)	0.6 (0.2)	0.499
Albumin (g/ml), mean (s.d)^a^ ^b^	3.8 (0.4)	3.6 (0.4)	3.9 (0.2)	0.094
CA19-9 (U/ml), mean (s.d)^a^ ^b^	241 (721)	74 (89)	377 (304)	0.087
CEA (ng/ml), mean (s.d)^a^ ^b^	73 (146)	53 (105)	72 (115)	1.000
Duration between the first and second hepatectomy (day), mean (s.d)^a^ ^b^	29 (22)	26 (14)	45 (46)	0.644
Extrahepatic metastasis at the diagnosis, *n* (%)				
Yes	7 (13.2)	3 (8.3)	4 (23.5)	0.127
No	46 (86.8)	33 (91.7)	13 (76.5)	
JHPBS nomogram score, mean (s.d)^a^ ^b^	16 (4)	14 (3)	19 (2)	0.007
Preoperative chemotherapy, *n* (%)				
Yes	42 (79.2)	25 (69.4)	17 (100.0)	0.010
No	11 (20.8)	11 (30.6)	0	
More than the second-line preoperative chemotherapy, *n* (%)				
Yes	29 (54.7)	19 (52.8)	10 (58.8)	0.237
No	13 (24.5)	6 (16.7)	7 (41.2)	
Not applicable	11 (20.8)	11 (30.6)	0 (0.0)	
Duration of preoperative chemotherapy, *n* (%)				
<3 months	10 (18.9)	6 (16.7)	4 (23.5)	0.972
≥3months	32 (60.4)	19 (52.8)	13 (76.5)	
Not applicable	11 (20.8)	11 (30.6)	0	
Radiological effect of preoperative chemotherapy, *n* (%)				
Partial response	23 (43.4)	16 (44.4)	7 (41.2)	0.311
Stable disease	15 (28.3)	8 (22.2)	7 (41.2)	
Unknown	4 (7.5)	1 (2.8)	3 (17.6)	
Not applicable	11 (20.8)	11 (30.6)	0	
The first hepatectomy and colon surgery at the same time, *n* (%)				
Yes	12 (22.6)	8 (22.2)	4 (23.5)	0.915
No	41 (77.4)	28 (77.8)	13 (76.5)	
Procedure of the first hepatectomy, *n* (%)				
PVE with partial hepatectomy	42 (79.2)	29 (80.5)	13 (76.5)	0.743
PVE with partial hepatectomy and RFA	5 (9.4)	4 (11.1)	1 (5.9)	
PVE with anatomical hepatectomy	4 (7.5)	2 (5.6)	2 (11.8)	
PVE with anatomical and partial hepatectomy	2 (3.8)	1 (2.8)	1 (5.9)	
Treating liver nodules number at the first hepatectomy, *n* (%)				
≤5	37 (69.8)	33 (91.7)	4 (23.5)	<0.001
>5	16 (30.2)	3 (8.3)	13 (76.5)	
Operation time in the first hepatectomy (min), mean (s.d)^a^ ^b^	351 (165)	329 (172)	519 (135)	<0.001
Blood loss in the first hepatectomy (ml), mean (s.d)^a^ ^b^	681 (955)	434 (457)	1527 (1984)	0.147
Complication after the first hepatectomy, *n* (%)				
Yes	4 (7.5)	3 (8.3)	1 (5.9)	0.753
No	49 (92.5)	33 (91.7)	16 (94.1)	
Hospital stays in the first hepatectomy (day), mean (s.d)^a^ ^b^	40 (27)	37 (22)	46 (34)	0.563
Procedure of the second hepatectomy, *n* (%)				
Liver lobectomy	41 (77.4)	28 (77.8)	13 (76.5)	0.562
Liver lobectomy with partial hepatectomy	9 (17.0)	5 (13.9)	4 (23.5)	
Liver lobectomy with RFA	2 (3.8)	2 (5.6)	0	
Liver lobectomy with partial hepatectomy and RFA	1 (1.9)	1 (2.8)	0	
Operation time in the second hepatectomy (min), mean (s.d)^a^ ^b^	350 (121)	336 (118)	337 (151)	0.239
Blood loss in the second hepatectomy (ml), mean (s.d)^a^ ^b^	866 (732)	742 (719)	1152 (1002)	0.960
Complication after the second hepatectomy, *n* (%)				
Yes	14 (26.4)	9 (25.0)	4 (23.5)	0.908
No	39 (73.6)	27 (75.0)	13 (76.5)	
Hospital stays in the second hepatectomy (day), mean (s.d)^a^ ^b^	19 (12)	22 (12)	18 (18)	0.960
Liver steatosis, *n* (%)				
Yes	15 (28.3)	8 (22.2)	7 (41.2)	0.261
No	18 (34.0)	13 (36.1)	5 (29.4)	
Unknown	20 (37.7)	15 (41.7)	5 (29.4)	
Sinusoidal dilatation in the liver, *n* (%)				
Yes	10 (18.9)	5 (13.9)	5 (29.4)	0.325
No	22 (41.5)	15 (41.7)	7 (41.2)	
Unknown	21 (39.6)	16 (44.4)	5 (29.4)	
Residual tumor, *n* (%)				
R0	31 (58.5)	21 (58.3)	10 (58.8)	0.900
R1	11 (20.8)	7 (19.4)	4 (23.5)	
R2	11 (20.8)	8 (22.2)	3 (17.6)	
Adjuvant therapy, *n* (%)				
Yes	23 (43.4)	17 (47.2)	6 (35.3)	0.413
No	30 (56.6)	19 (52.8)	11 (64.7)	
Observation period after the first hepatectomy (month), mean (s.d)^a^	21.8 (20.4)	18.0 (17.8)	18.3 (16.8)	0.402
Disease progression after TSH, *n* (%)				
Yes	31 (58.5)	19 (52.8)	12 (70.6)	0.219
No	22 (41.5)	17 (47.2)	5 (29.4)	

Values are *n* (%) unless indicated otherwise indicated.

^a^ Mean (s.d.).

^b^ Missing data includes as follows: Body mass index for 4 patients, ICGR15 for 8, Prothrombin Time for 5, Total Bilirubin for 5, Albumin for 6, CA19-9 for 17, CEA for 20, Duration between the first and second liver surgery for 11, JHPBS score for 17, Operation time in the first liver surgery for 3, Blood loss in the first liver surgery for 7, Hospital stays in the first liver surgery for 11, Operation time in the second surgery for 8, Blood loss in the second liver surgery for 8 and Hospitals stay in the second liver surgery for 18.

ASA-PS, American Society of Anesthesiologist’s Physical Status; CA19-9, carbohydrate antigen 19-9; CEA, carcinoembryonic antigen; ICGR15, indocyanine green retention rate at 15 min; JHPBS, Japanese Society of Hepato-Biliary-Pancreatic Surgery; PVE, portal vein embolization; RFA, radio frequency ablation; TSH, two-stage hepatectomy.

Twelve patients (12/53, 22.6%) required blood transfusions during first or second hepatectomy. Four patients had complications after the first hepatectomy (7.5%) as follows: 3 had bile leakage and one had small bowel obstruction. Fourteen patients had complications after the second hepatectomy (26.4%) as follows: 4 had bile leakage, 4 had wound infection, 3 had intra-abdominal abscess, 2 had pleural effusion and one had hyperbilirubinemia. There were no in-hospital mortalities.

**Figure 2:**
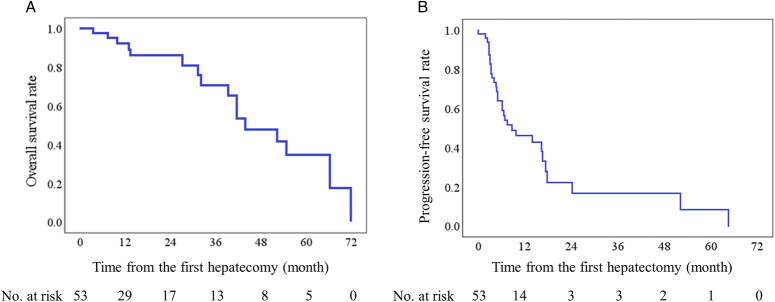
Overall and progression-free survival in patients undergoing two-stage hepatectomy for colorectal liver metastasis. (A) Overall survival, (B) progression-free survival.

The median follow-up period was 13.4 months, with a range from 1.0 to 71.7 months. The mean observation period was 21.8 ± 20.4 months. The observation period in patients treated in late years was shorter than that in early years (mean: 47.1 vs. 19.1, months). During the observation period, 16 of 53 patients died of cancer (30.2%), 31 of 53 (58.5%) had disease progression after TSH including 18 had tumor recurrence after R0 resection. The estimated OS and PFS rate for all patients at 5-year were 34.7%, and 8.3%, respectively (Figs.2A, B). The mean estimated OS and PFS for the entire cohort were 46.4 and 17.6 months, respectively.

To determine the optimal cut-off values of total number of liver nodules for predicting survival, ROC curve was shown in Supplementary Figure 1, Supplemental Digital Content 2, http://links.lww.com/JS9/C746. An area under the ROC curve was 0.658. The optimal cut-off was identified as more than 10 liver nodules, based on maximizing the combined sum of sensitivity (56%) and specificity (81%). The determination of cut-off values for other continuous variables was conducted in a similar manner.

Table 2 showed univariate and multivariate analyses for OS in patients undergoing TSH for CRLM. Univariate analyses showed that a number of liver nodules (>10 vs. ≤ 10) and lymphatic invasion of the primary tumor (ly3, ly2 vs. ly1, ly0) were associated with OS. Using variables with *P* value less than 0.05 in the univariate analysis, multivariate analyses revealed that number of liver nodules (>10 vs. ≤ 10) (HR 4.284; 95% CI 1.224–14.99, *P*= 0.023) was significantly associated with OS. As shown in Table 1, patients with more than 10 liver nodules were younger (*P*= 0.015), had higher JHPBS nomogram scores (*P*= 0.007), and were more likely to receive preoperative chemotherapy (*P*= 0.010) compared to those with fewer than 10 liver nodules.

**Table 2:** Univariate and multivariate analyses using Cox hazard model for overall survival in patients undergoing two-stage hepatectomy using portal vein embolization for colorectal liver metastasis.

	Univariate		Multivariate	
Characteristics	HR (95% CI)	*P*	HR (95% CI)	*P*
Age (>65/ ≤ 65, year)	0.332 (0.041–2.657)	0.298		
BMI (<23.5/ ≥ 23.5, kg/m^2^)	0.381 (0.649–3.285)	0.381		
Sex (Male/female)	0.444 (0.147–1.340)	0.150		
ASA-PS (Class 2, 3/Class 1)	0.444 (0.198–2.032)	0.635		
Timing of liver metastasis (synchronous/metachronous)	1.000 (0.001–444385)	1.000		
Total number of liver nodules (>10/ ≤ 10)	5.525(1.694–18.01)	0.005	4.284 (1.224–14.99	0.023
Largest tumor diameter (>3.5/ ≤ 3.5, cm)	0.963 (0.324–2.857)	0.946		
Primary tumor site (rectum/colon)	1.120 (0.349–3.596)	0.849		
Tumor differentiation of primary tumor (others/well)	2.508 (0.555–11.33)	0.232		
T factor of primary tumor (T4/T3, T2)	1.148 (0.308–4.277)	0.837		
Lymph node metastasis of primary tumor (N2/N1, N0)	1.446 (0.492–4.429)	0.487		
Lymphatic invasion of primary tumor (ly3, ly2/ly1, ly0)	3.207 (1.060–9.708)	0.039	1.991 (0.599-6.618	0.261
Venous invasion of primary tumor (v3, v2/v1, v0)	1.305 (0.456–3.738)	0.620		
KRAS mutation of primary tumor (yes/no)	2.227 (0.628–7.895)	0.215		
ICGR15 (>10/ ≤ 10, %)	1.879 (0.574–6.145)	0.297		
Prothrombin time (<100/ ≥ 100, %)	0.703 (0.300–1.649)	0.418		
Total bilirubin (>0.8/ ≤ 0.8, mg/dl)	2.683 (0.476–15.12)	0.263		
Albumin (<4.0/ ≥ 4.0, g/ml)	0.577 (0.196–1.703)	0.320		
CA19-9 (>100/ ≤ 100, U/ml)	1.345 (0.403–4.405)	0.630		
CEA (>20/ ≤ 20, ng/ml)	0.557 (0.138–2.254)	0.412		
Extrahepatic metastasis (yes/no)	1.341 (0.367–4.902)	0.657		
JHPBS nomogram score (>18/ ≤ 18)	2.214 (0.643–7.622)	0.207		
Preoperative chemotherapy (yes/no)	3.820 (0.847–17.22)	0.081		
More than the second-line preoperative chemotherapy (yes/no)	2.643 (0.839–8.325)	0.097		
Duration of preoperative chemotherapy ( ≥ 3/<3, month)	1.086 (0.292–4.040)	0.902		
Radiological effect of preoperative chemotherapy (stable disease/ partial response)	0.500 (0.137–1.831)	0.296		
Combined colon with hepatectomy at the first surgery (yes/no)	1.111 (0.381–3.238)	0.848		
Operation time of the first hepatectomy (>240/ ≤ 240, min)	6.370 (0.829–48.96)	0.075		
Blood loss of the first hepatectomy (>500/ ≤ 500, ml)	0.986 (0.296–3.286)	0.982		
Complication after the first hepatectomy (yes/no)	2.475 (0.538–11.38)	0.245		
Operation time of the second hepatectomy (>420/ ≤ 420, min)	0.617 (0.198–2.025)	0.426		
Blood loss of the second hepatectomy (>1000/ ≤ 1000, ml)	0.771 (0.231–2.572)	0.672		
Complication after the second hepatectomy (yes/no)	1.710 (0.566–5.163)	0.341		
Residual tumor (R2/R1, R0)	1.583 (0.433–5.783)	0.487		
Adjuvant therapy (yes/no)	0.968 (0.333–2.811)	0.952		

Values are in hazard ratio (HR) and 95% CI.

ASA-PS, American Society of Anesthesiologist’s Physical Status; CA19-9, carbohydrate antigen 19-9; CEA, carcinoembryonic antigen; ICGR15, indocyanine green retention rate at 15 min; JHPBS, Japanese Society of Hepato-Biliary-Pancreatic Surgery.

**Figure 3:**
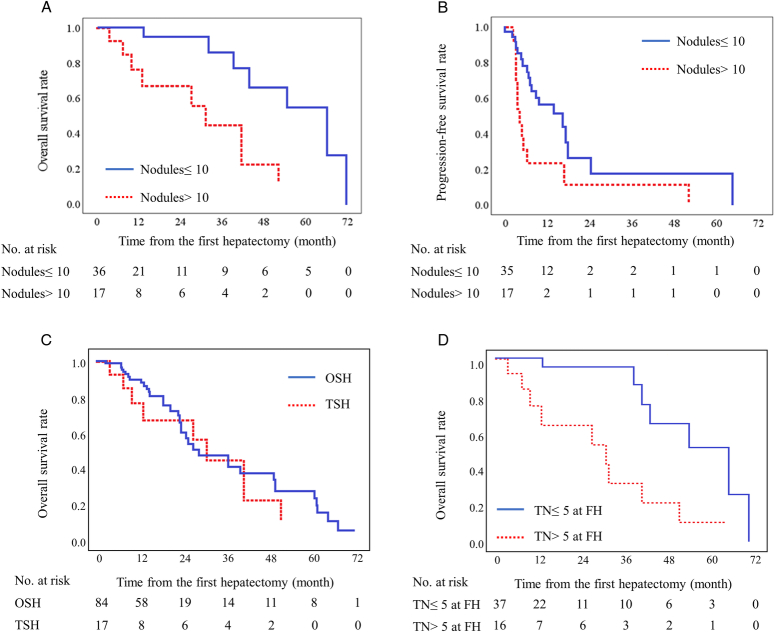
Overall and progression-free survival in patients undergoing two-stage hepatectomy (TSH) for colorectal liver metastasis according to 10 liver nodules. (A) Overall survival, (B) progression-free survival. (C) Overall survival in patients with initially borderline resectable or unresectable liver disease who have more than 10 nodules according to one-stage hepatectomy (OSH) and two-stage hepatectomy (TSH). (D) Overall survival in patients undergoing TSH for colorectal liver metastasis according to number of treated nodules (TN) at the first hepatectomy (FH).

More than 10 liver nodules were significant associated both OS (*P*= 0.001) and PFS (*P*= 0.017) (Fig. 3A & B). The estimated 5-year OS and PFS rates for patients with fewer than 10 liver nodules were 54.8% and 17.5%. The mean estimated OS and PFS for patients with fewer than 10 liver nodules were 55.8 and 20.7 months. The estimated 5-year OS and PFS rate for patients more than 10 liver nodules were 11.1% and 0.0%. The mean estimated OS and PFS for patients with more than 10 liver nodules were 30.2 and 10.8 months.

To examine whether more than 10 liver nodules was a specific predictor for TSH or not, we additionally acquired the information of 84 patients who undergo one-stage hepatectomy (OSH) for initially borderline resectable or unresectable CRLM which was more than 10 liver nodules from the database. We compared OS between the TSH group with the OSH group. No significant difference in OS was found between the two groups (Fig. 3C, *P*= 0.455), suggesting that more than 10 liver nodules is not a specific predictor for TSH. Furthermore, we examined an association between OS and treated liver nodules number at the first hepatectomy. Treating more than 5 liver nodules at the first hepatectomy is significantly associated with a worse prognosis (Fig. 3D, *P*< 0.001). Treating more than 5 liver nodules at the first hepatectomy is also significantly associated with a total number of liver nodules more than 10 (Table 1, *P*< 0.001).

**Figure 4:**
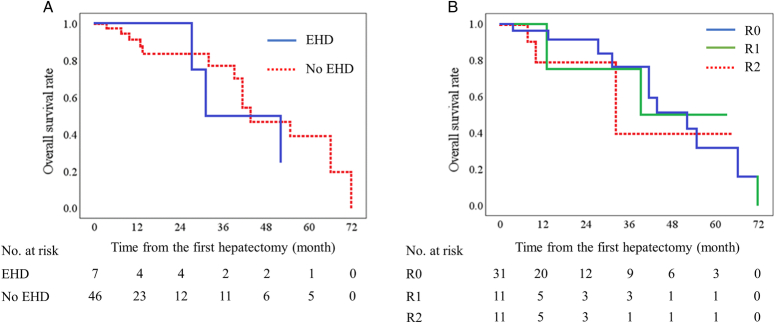
Overall survival in patients undergoing two-stage hepatectomy for colorectal liver metastasis according to (A) extrahepatic disease (EHD) and (B) the degree of residual tumor (R0, R1 and R2 resection).

Kaplan–Meier method and Log-rank test revealed that extrahepatic disease and residual tumor were not associated with OS in patients who underwent TSH for CRLM (Figs. 4A, B, *P=* 0.656, *P*= 0.782). The estimated 5-year OS rate was 25.0% for patients with extrahepatic disease and 38.9% for those without. The mean estimated OS was 40.6 months for patients with extrahepatic disease was 40.6 and 47.3 months for those without. On the other hand, the estimated 5-year OS rate for R0, R1 and R2 resection were 31.7%, 50.0%, and 39.4%. The mean estimated OS in R0, R1 and R2 resection was 48.0, 44.5 and 39.9 months.

Adjuvant therapy was conducted in 23 of 53 patients (43.4%) after the second liver surgery. The regimen of adjuvant therapy was as follows: FOLFOX-based therapy in 10 patients, 4 patients of FOLFIRI-based therapy, CapeOX in 3, UFT/LV in 2, other regimens in 3 and one patient had unavailable data. Adjuvant therapy was not associated with OS and PFS regardless of total number of liver nodules (Supplementary Figure 2, Supplemental Digital Content 2, http://links.lww.com/JS9/C746)

In the entire cohort, 31 patients had disease progression after TSH as follows: 13 had intrahepatic disease, 7 had extrahepatic disease and 11 had both in Supplementary Table 2, Supplemental Digital Content 2, http://links.lww.com/JS9/C746. While 15 patients underwent chemotherapy and 2 selected palliative care for disease progression after TSH, 14 received repeat hepatectomy for intrahepatic diseases. In the repeat hepatectomy group, 4 patients had concomitant extrahepatic diseases, and their treatment contained both surgery and chemotherapy. Supplementary Table 3, Supplemental Digital Content 2, http://links.lww.com/JS9/C746 showed that the repeat hepatectomy group were most likely to have the pattern of intrahepatic progression compared to the chemotherapy group (10 vs. 3, *P*= 0.008), and the number of liver nodules in the repeat hepatectomy group was significantly less than that in the chemotherapy group (mean 1.9 vs. 4.1, *P*= 0.020). Repeat hepatectomy for disease progression after TSH was superior to chemotherapy in terms of survival starting from disease progression (Supplementary Figure 3, Supplemental Digital Content 2, http://links.lww.com/JS9/C746, *P*< 0.001). The mean estimated survival for the repeat hepatectomy group and the chemotherapy group was 49.6 and 18.7 months, respectively.

## Discussion

In this study, the estimated 5-year survival rate for patients undergoing TSH was 34.7%, aligning with that of previous studies (23.0–44.2%)^12–15^. Given that 5-year survival rate for patients who have metastatic CRLM without hepatectomy is 7.0–26.0%^23,24^, our finding suggests that TSH is a more effective treatment option for Japanese patients with borderline resectable and unresectable CRLM. It is feasible to convert to surgery in initially unresectable CRLM because the rate of conversion surgery has reported to be 23.1–56.9%^25–27^. Conversion surgery improves survival in patients who are diagnosed with initially unresectable CRLM compared to patients without hepatectomy (median survival: 54–77 vs. 21–28, months)^25,27^. Even if CRLM is initially evaluated as unresectable, these previous studies indicate that the timing of conversion to surgery including TSH should be considered.

Twelve patients underwent extra surgical procedures in addition to lobectomy at the second hepatectomy (Table 1), indicating potential disease progression in the remnant liver after the first hepatectomy with PVE. In CRLM, PVE doesn’t affect prognosis but leads to disease progression in 66% of cases^28^. Previous studies revealed the molecular association between PVE and disease progression of CRLM^29^. Expression of cytokines, growth factors and tumor proliferation following PVE were facilitated in case of disease progression compared to that before PVE^29^. These previous studies suggest that confirming disease progression should be considered before the second hepatectomy.

Our finding shows that having more than 10 liver nodules increases the mortality risk by 4.0-fold compared to those with fewer than 10 liver nodules (Table 2). The comparison between TSH and OSH revealed that having more than 10 liver nodules was not a specific predictor for survival in patients with TSH (Fig. 3c). The number of liver nodules has been reported to be a survival predictor in patients with CRLM who undergo hepatectomy^30–32^. While previous studies have reported that having more than 6 liver nodules was as independent prognostic factor in patients undergoing TSH for unresectable CRLM^11,15^, the cut-off value of liver nodules in our study is larger than that in previous studies.

The estimated 5-year survival rate for patients with fewer than 10 liver nodules was 54.8%, suggesting that the effectiveness of TSH might be limited for CRLM up to 10 liver nodules at the present. Unfortunately, survival outcome for patients with more than 10 liver nodules was only 11.1%, which is worse compared to those received initial chemotherapy for unresectable metastatic CRC (11.1% vs. 20.0%)^33^. Additionally, treating more than 5 liver nodules at the first hepatectomy was also significantly associated with a total of more than 10 liver nodules (Table 1). Tumor progression in the remnant liver at the first hepatectomy may be associated with worse prognosis in patients with more than 10 liver nodules. However, our small study population (*n*=53) and short observation period (mean: 21.8 months) might underestimate survival in these patients. Further large studies with long-term observation would be required to address these biases.

Additionally, adjuvant therapy might not improve surgical outcomes in these patients (Figure S1). In parallel with advancements in surgical technique, precision chemotherapies based on gene profiling or oncological signal pathway in CRC has gradually developed over the decade^34^. These precision medicines might be expected for improving the survival of patients undergoing TSH for CRLM more than 10 liver nodules.

Unexpectedly, extrahepatic metastasis and residual tumor were not associated with OS in patient who undergoing TSH for CRLM (Table 2, Fig. 4). Previous studies have reported that these characteristics are survival predictors for patients undergoing hepatectomy for CRLM^32,35^. Nonetheless, systematic review has noted that hepatectomy could be a salvage treatment for patients with both of intrahepatic and extrahepatic metastasis, especially for patients with liver and lung metastasis^36^. Other studies suggested that preoperative chemotherapy or adjuvant therapy might bridge the OS gap between R0 and R1 resection^37,38^. The estimated 5-year survival rate for patients with extrahepatic disease, R1 and R2 resection in our study was superior to that for patients who received initial chemotherapy for metastatic CRC (25.0%, 50.0%, 39.4% vs. 20.0%)^3,33^. This comparison suggested that TSH might be a salvage treatment, even in cases of extrahepatic disease or R1 and R2 resection.

In planning TSH, the second hepatectomy will be hesitated in case of disease progression during preoperative chemotherapy, as well as disease progression in the remnant liver following the first hepatectomy. However, our study didn’t have disease progression during preoperative chemotherapy due to selection bias. Even if disease progression during preoperative chemotherapy is resectable, hepatectomy is recommended rather than chemotherapy alone because 3-year survival rate for repeat hepatectomy is superior to that for chemotherapy alone (49.3% vs. 10.6%)^39^. On the other hand, a retrospective study highlighted that patients undergoing hepatectomy for disease progression in the FLR exhibited improved survival outcome compared to those who dropped out from TSH (median survival 42 months vs. 26 months)^13^. This previous finding suggests that resectable disease progression in the remnant liver should not be considered as surgical contraindications for the second hepatectomy.

In our study, five patients underwent a laparoscopic approach for TSH was conducted. Recent advancements in laparoscopic equipment and techniques have led to an increase in laparoscopic approach for TSH^40,41^. The comparison between open and laparoscopic TSH reported laparoscopic TSH didn’t affect long-term survival, while it reduced complications and decreased both the hospital stay in the first and second hepatectomy as well as the interval from surgery to adjuvant therapy^42^. Furthermore, laparoscopic surgery provides less adhesion compared to open surgery^43^, potentially making repeat hepatectomy following TSH easier. These advantages will lead to an increase in laparoscopic TSH in the future. However, laparoscopic liver lobectomy is more complex procedure and requires a comprehensive experience^41^. Moreover, PVE induced significant peri-portal inflammation, potentially making the laparoscopic dissection of the hilar part and liver parenchyma more difficult at the second hepatectomy^41^. These factors imply that laparoscopic TSH requires skilled laparoscopic techniques and should be limited to expert centers.

Our result showed that repeat hepatectomy for disease progression exhibited superior outcomes compared to chemotherapy in patients undergoing TSH (Supplementary Figure 2, Supplemental Digital Content 2, http://links.lww.com/JS9/C746). Repeat hepatectomy is recommended for intrahepatic recurrence in patients with CRLM, because 5-year survival rate for repeat hepatectomy was 31 to 52%^44–47^. In addition, two studies have reported that surgery for recurrent diseases following TSH offered a favorable prognosis compared to other treatments^14,18^. These studies support our finding, suggesting that repeat surgery may be a salvage treatment for disease progression after surgery even if patients had received TSH.

This study has several limitations due to its retrospective nature, including variations in preoperative chemotherapy and adjuvant therapy among patients, which can potentially influence the survival analysis. Molecular-targeted therapy for CRC in Japan was less common during 2005–2007, leading to multiple groups with different treatment backgrounds. The short observation period (mean: 21.8 months, Table 1) may reduce the reliability of our result, and it can lead to underestimations or overestimations of treatment outcomes. Given these limitations, we calculated the estimated mean survival period and 5-year survival rate. Our study population was small due to the limited patients who might meet TSH criteria and the likelihood that TSH had not yet widely adopted in Japan during the data collection period. This small study population might affect the generalizability of our findings. There was potential selection bias of patients in patient’s status and controllable disease at each institution, since only one patient complicated severe systemic disease (ASA-PS class 3) and most patients exhibited PR or SD after preoperative chemotherapy. Previous researches suggest that chronic viral hepatitis may increase the risk of CRLM and unfavorable prognosis^48,49^, but our study was unable to assess the impact of chronic viral infections on postoperative outcomes due to the absence of cases with viral hepatitis. Additionally, we lacked information on patients who dropped out from TSH, future remnant liver volume and macro-vascular invasion, which are crucial for clinical management. Overcoming these limitations may require a randomized clinical trial or a larger cohort study with extended observation periods.

In conclusion, TSH was confirmed to be a safety procedure with acceptable survival outcomes in the Japanese cohort. More than 10 liver nodules might predict unfavorable outcomes for patients with CRLM undergoing TSH. While adjuvant therapy might be insufficient to improve their surgical outcome, TSH could be a salvage option for controllable extrahepatic disease or R1 and R2 resection. Additionally, repeat hepatectomy could be a salvage treatment for resectable intrahepatic recurrence following TSH.

## Ethical approval

This study was conducted with the approval of the institutional review board (approval number, 2022-003a; Dokkyo Medical University).

## Consent

An opt-out method was used to obtain patient consent.

## Source of funding

This research did not receive support from any sources of funding.

## Author contribution

T.S.: conceptualization, data curation, formal analysis, writing—original draft. T.A.: supervision, methodology, writing—review and editing. M.I.: supervision, writing—review and editing. K.S.: supervision, writing—review and editing. T.B.: supervision, writing—review and editing. G.H.: supervision, writing—review and editing. K.K.: supervision, writing—review and editing. M.Y.: supervision, writing—review and editing. K.T.: supervision, writing—review and editing. I.E.: supervision, writing—review & editing. K.H.: supervision, writing—review and editing. M.I.: supervision, writing—review and editing. Y.H.: supervision, writing—review and editing. Y.K.: supervision, writing—review and editing. S.K.: supervision, writing—review and editing. T.Y.: supervision, writing—review and editing. S.N.: supervision, writing—review and editing. K.T.: supervision, writing—review and editing. H.K.: supervision, writing—review and editing. K.Y.: supervision, writing—review and editing. K.T.: supervision, writing—review and editing. S.M.: supervision, writing—review and editing. M.M.: supervision, writing—review and editing. K.S.: supervision, writing—review and editing. Y.A.: supervision, writing—review and editing.

## Conflicts of interest disclosure

The authors declare no conflict of interest.

## Research registration unique identifying number (UIN)

Registry used: UMIN-CTR

Registration ID: UMIN000053401

https://center6.umin.ac.jp/cgi-open-bin/ctr/ctr_view.cgi?recptno=R000060945

## Guarantor

Takayuki Shimizu and Taku Aoki are the guarantor of the present study.

## Data availability statement

The data provided here are accurate to the best of our knowledge.

## Provenance and peer review

This paper was not invited.

## Assistance with the study

The authors express their gratitude for the contributions of the physicians from 148 departments and 145 institutions who diligently registered patients with colorectal liver metastases in this registry.

## Supplementary Material

SUPPLEMENTARY MATERIAL
