# Supplementary material for: Evaluation of two-stage hepatectomy using portal vein embolization for colorectal liver metastasis: a retrospective nationwide cohort survey in Japan
Source: Int J Surg. 2024 Jun 13;110(10):6691–701. doi: 10.1097/JS9.0000000000001811 (PMC11486952; doi:10.1097/JS9.0000000000001811)
Supplement: SUPPLEMENTARY MATERIAL [file js9-110-6691-s002.docx]

**Supplementary Table 1. Association between underlying dieases and number of liver nodules**

|  | Entire cohort  (n=53) | Number of nodules≤ 10  (n=36) | Number of nodules> 10  (n=17) | *P*-value |
| --- | --- | --- | --- | --- |
| ASA-PS |  |  |  |  |
| Class 1 | 29 (54.7) | 19 (52.8) | 10 (58.8) | 0.749 |
| Class 2 | 23 (43.4) | 16 (44.4) | 7 (41.2) |  |
| Class 3 | 1 (1.9) | 1 (2.8) | 0 (0.0) |  |
| Viral hepatitis |  |  |  |  |
| Yes | 0 (0.0) | 0 (0.0) | 0 (0.0) | 1.000 |
| No | 53 (100.0) | 36 (100.0) | 17 (100.0) |  |

Values are parentheses and percentages

**Supplementary Table 2. Association between disease progression pattern and number of liver nodules**

| Disease progression pattern | Entire cohort  (n=31) | Number of nodules< 10  (n=19) | Number of nodules> 10  (n=12) | *P*-value |
| --- | --- | --- | --- | --- |
| Intrahepatic progression | 13 (41.9) | 10 (52.6) | 3 (25.0) | 0.328 |
| Extrahepatic progression | 7 (22.6) | 3 (15.8) | 4 (33.3) |  |
| Intrahepatic and extrahepatic progression | 11 (35.5) | 6 (31.6) | 5 (41.7) |  |

Values are parentheses and percentages

**Supplementary Table 3. Association between therapy and disease progression after surgery**

| Characteristics | Repeat hepatectomy (n=14) | Chemotherapy  (n=15) | *P*-value |
| --- | --- | --- | --- |
| Progression pattern |  |  |  |
| Intrahepatic progression | 10 (71.4) | 3 (20.0) | 0.008 |
| Extrahepatic progression | 0 (0.0) | 5 (33.3) |  |
| Intrahepatic and extrahepatic progression | 4 (28.6) | 7 (46.7) |  |
| Number of intrahepatic progression, mean (s.d)* | 1.9 (1.4) | 4.1 (2.8) | 0.020 |

Values are n (%) unless indicated otherwise; values are *mean(s.d.). Data was missing in 6 patients for Number of intrahepatic progression.

**Supplementary Figure 1. Receiver operating characteristics (ROC) curve** **of total number of liver nodules for predicting survival after surgery.**


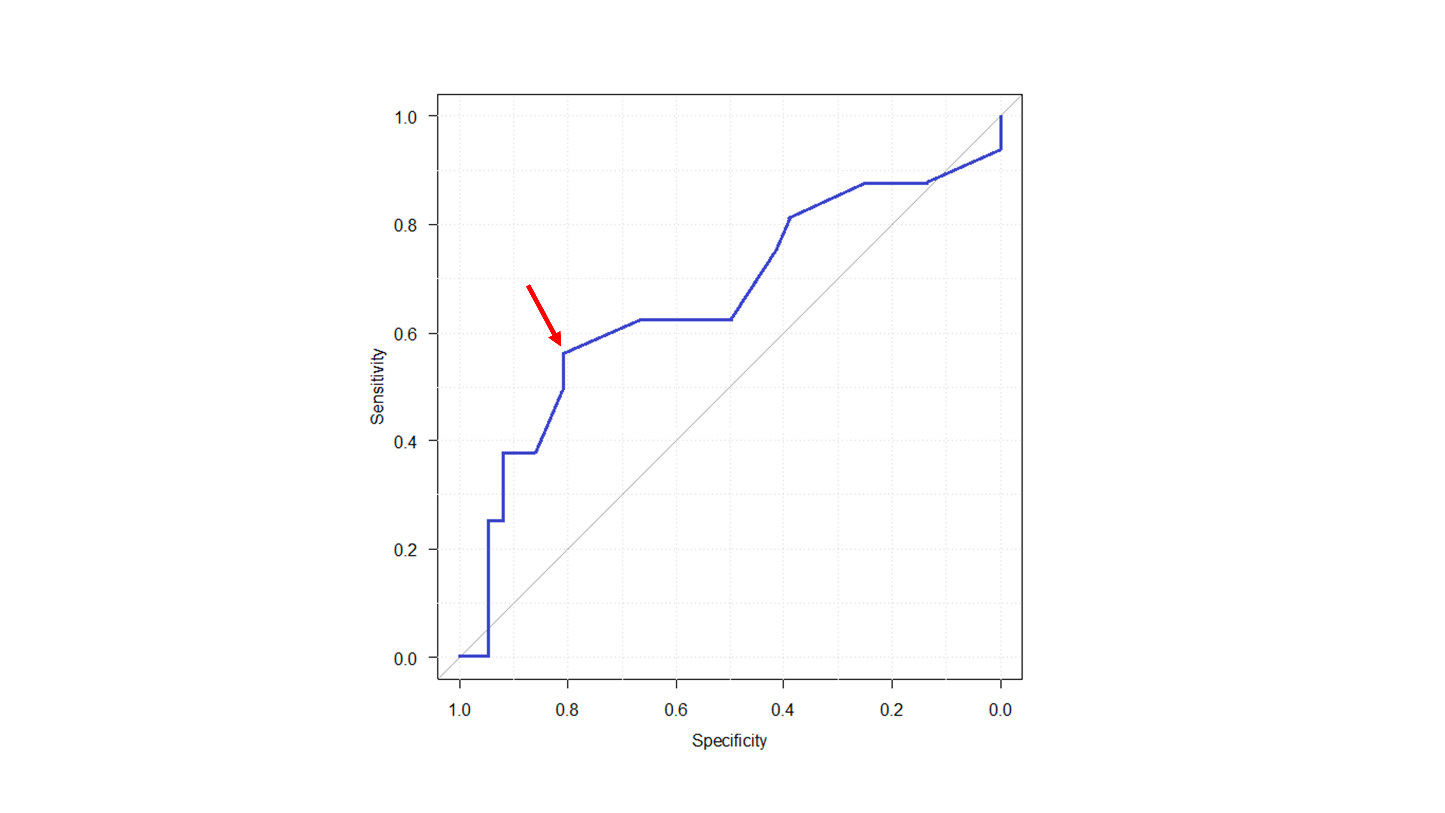


An area under the ROC curve was 0.658. The optimal cut-off was identified as more than 10 liver nodules, based on maximizing the combined sum of sensitivity (56%) and specificity (81%).

**Supplementary Figure 2. Overall and progression-free survival in patients undergoing two-stage hepatectomy for colorectal liver metastasis according to adjuvant therapy**


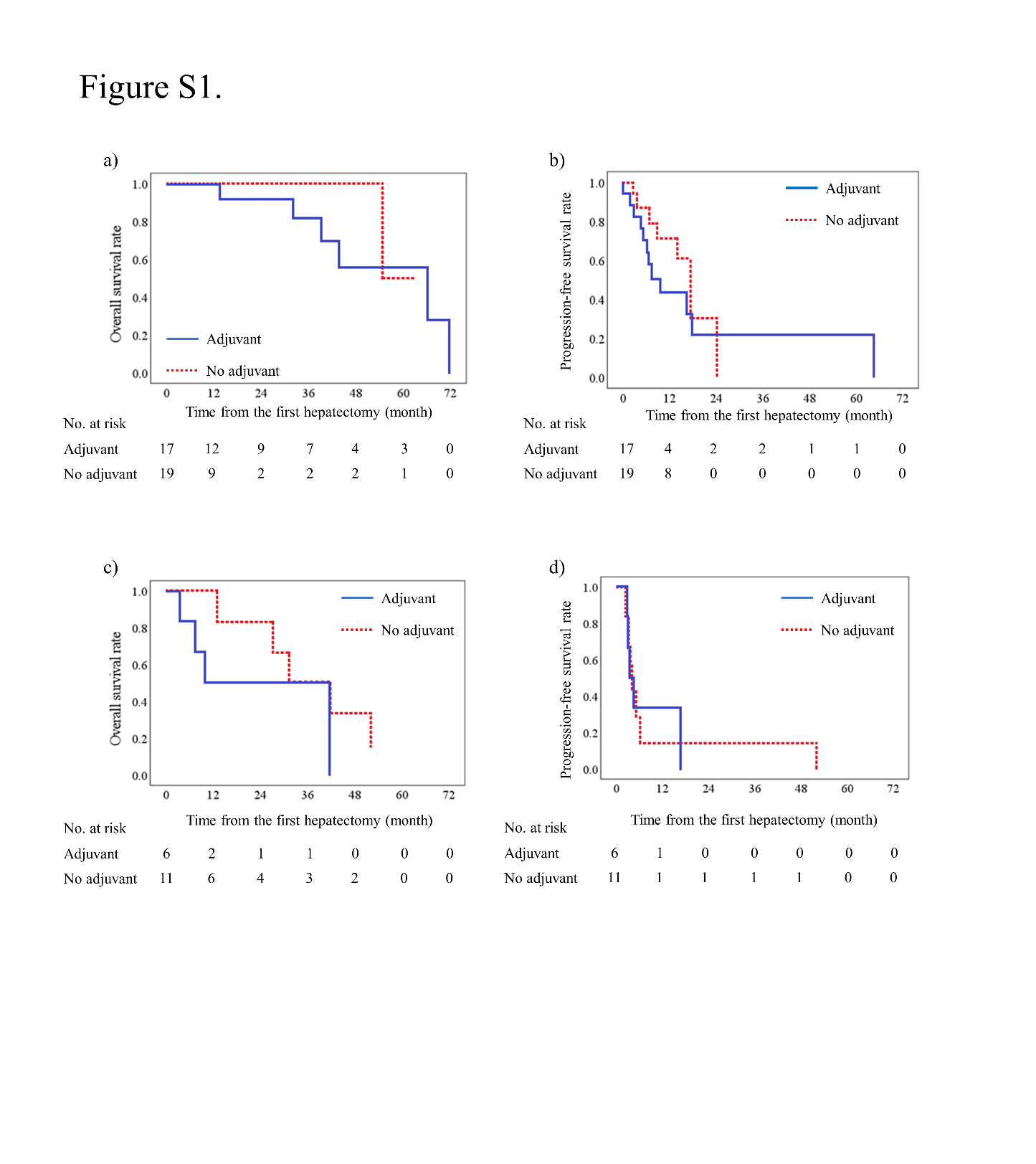


a) Overall survival in patients with liver nodules ≤10 (*P*= 0.670), b) Progression-free survival in patients with liver nodules ≤10 (*P*= 0.397), c) Overall survival in patients with liver nodules >10 (*P*= 0.233), d) Progression-free survival in patients with liver nodules >10 (*P*= 0.909).

**Supplementary Figure 3. Overall survival in patients with repeat hepatectomy and chemotherapy for progression disease progression after two-stage hepatectomy for colorectal liver metastasis**


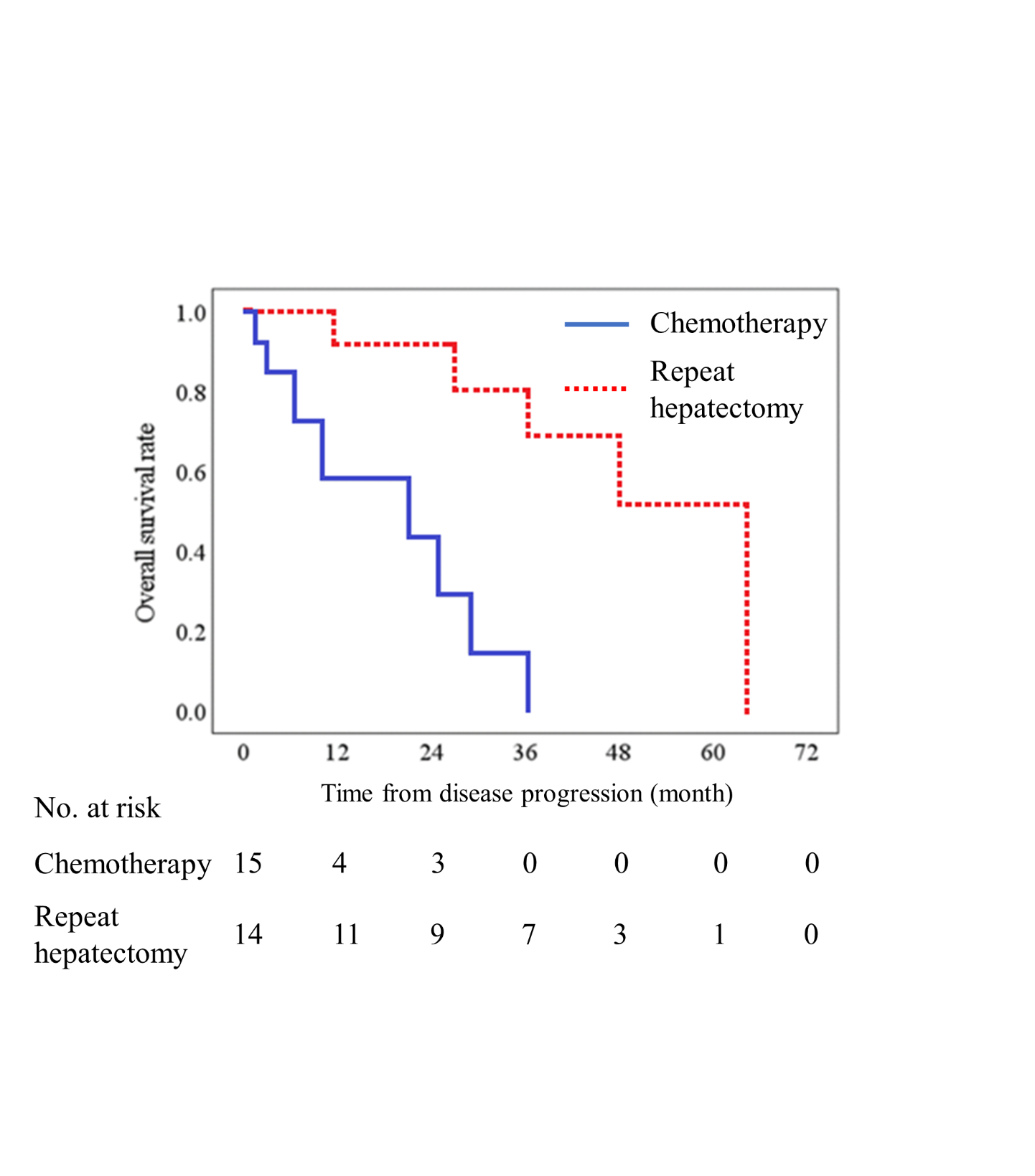


Kaplan-Meier method and log-rank test revealed that Repeat hepatectomy for disease progression after two-stage hepatectomy was superior to chemotherapy in overall survival time from disease progression (*P*< 0.001)
